# Treatment Dilemmas in a Young Man Presenting with Narcolepsy and Psychotic Symptoms

**DOI:** 10.1155/2011/804357

**Published:** 2011-11-28

**Authors:** Victoria Fernandez, Sharon Davies, Nicola Walters

**Affiliations:** ^1^Springfield University Hospital, South West London and St. George'ss Mental Health Trust, 61 Glenburnie Rd., London SW17 7DJ, UK; ^2^Academic Unit of C&A Psychiatry, Imperial College London, St. Mary's Campus, Norfolk Place, Paddington, London W2 1PG, UK; ^3^The Wells Unit, West London Mental Health Trust, Uxbridge Road Southall, London UB1 3EU, UK; ^4^Respiratory and General Medicine, St. George's Hospital, St. George's Healthcare NHS Trust, Blackshaw Rd., London SW17 0QT, UK

## Abstract

Psychotic features can be present in both narcolepsy and psychosis, which can result in challenges in diagnosis and management. The prevalence of both conditions is low and the reports in young people are scarce. Our report illustrates the relevance of a thorough differential diagnosis as well as the need to explore treatment avenues based on the evidence available for both narcolepsy and psychosis symptoms to try and maximise the therapeutic impact.

## 1. Introduction

Narcolepsy is a chronic neuropsychiatric disorder characterized by severe, irresistible daytime sleepiness and symptoms described as rapid eye movement (REM) sleep-related symptoms, including sudden loss of muscle tone (cataplexy), hypnagogic hallucinations, and sleep paralysis. Narcolepsy was considered a disorder of adulthood, but accumulating evidence indicates that narcolepsy may start during childhood. However, the precise prevalence of hypersomnia in children is unknown [1]. Narcolepsy and psychosis can share common features and age of onset, leading to challenges for diagnosis and subsequent management, as illustrated in case reports [2–8] and case control studies [9, 10]. These look at adults [2–10], with anecdotal references to younger patients [11]. We report on a 17-year-old man with narcolepsy complicated by psychotic symptoms and the therapeutic approach followed.

## 2. Case Report

A 17-year-old man of Chinese ethnicity was admitted to our unit in 2009 after presenting with auditory and somatic hallucinations and paranoid delusions, which resulted in an incident of aggression at home. He had a comorbid diagnosis of narcolepsy.

### 2.1. History of Narcolepsy

In 2007 the patient had been diagnosed with narcolepsy and cataplexy after a history of recurrent episodes of loss of muscle tone related with high emotions (i.e., strong laughter) and daytime somnolence. The diagnosis had initially been made on clinical grounds following the ICSD (International Classification of Sleep Disorders) criteria B and C (B = Recurrent daytime naps for >3 months and C = Sudden bilateral loss of muscle tone in association with intense emotion (cataplexy)) [12]. Polysomnography (PSG) had not been considered necessary at that stage nor Human Leukocyte Antigen (HLA) typing as studies on Hong Kong Chinese narcoleptics [13] had found out that all patients were HLA DR2- and DQ1-positive so the view then was that it would not provide any additional relevant information for the diagnosis. He was receiving followup from a sleep disorder service and was not on any medication for narcolepsy or cataplexy. 

While in our unit, arranging further tests (i.e., PSG, Multiple Sleep Latency Testing-MSLT) was considered but put on hold due to the young man's mental health deterioration.

### 2.2. History of Psychotic Episodes

The patient had several prior admissions to psychiatric wards in 2008 and 2009.

During his admission in 2008 his psychotic symptoms appeared to be present mostly at bedtimes in the form of hypnagogic and hypnopompic hallucinations with little evidence of psychosis during the day. The opinion then was that the underlying narcolepsy could explain his psychotic experiences so his antipsychotic medication (amisulpride) was subsequently reduced without any evidence of deterioration in his mental state. 

In his admission in 2009 his presentation appeared different, with paranoid symptoms and auditory hallucinations, which responded to haloperidol. However, due to complaints of sleepiness the medication was changed to aripiprazole. His compliance was erratic, with clear deterioration of his symptoms when noncompliant. At discharge he was given a diagnosis of hebephrenic schizophrenia.

### 2.3. Physical History and Other Additional Investigations

In 2008 a WISC-IV (Wechsler Intelligence Scale for Children, version IV) was completed to clarify the nature of his difficulties retaining information. The result suggested that this was not related to his cognitive abilities, which were in the low-average range.

In 2009 he had an MRI (Magnetic Resonance Imaging) of the brain and EEG (Electroencephalogram) that were both reported as normal.

In his admission to our unit in 2010, input from Speech and Language Therapy was sought to assess the fact that his mother tongue was Chinese, which caused the subsequent cross-cultural barrier and the possible impact of this on his presentation and interpretation of his symptoms. This assessment did not suggest that his language or cultural factors were contributing factors to the described clinical picture. In addition, his physical examination, blood tests, and toxicological screening did not exhibit significant alterations.

### 2.4. Progress in Our Unit

His medication was increased from 4 mg/d to 6 mg/d risperidone with incomplete symptom resolution. He continued to experience auditory hallucinations and showed incongruent affect. He was impaired in his functioning, particularly in his thought processing. As in his admission in 2008, some of his psychotic symptoms (i.e., tactile and somatic hallucinations) seemed to be only present around sleeping times. His sleep was disturbed at the night (i.e., shouting, singing, or responding to voices whilst asleep) and following the awakening either from night sleep or frequent naps in relation to his daytime sleepiness, he had substantial difficulties to process and respond to verbal information. It is possible that these behaviours were the result of REM sleep behaviour disorder (RBD). There is evidence in the literature that RBD is present in up to a third of patients with narcolepsy [14].

To try to clarify the clinical picture, in consultation with his consultant at the Sleep Disorder Clinic, we started him on a trial of 7.5 mg/d zopiclone, which proved effective to stop the described night-time somatic hallucinations. As daytime sleepiness and marked difficulties in concentration and thought process were still quite noticeable, a dose of 200 mg/d modafinil was tried with significant positive impact on concentration and daytime general function. Significant thought disorder and distraction remained so a trial of Clozapine was started. Unfortunately, it had to be discontinued due to adverse haematological results.

## 3. Discussion

Narcolepsy can be related to psychosis in three ways [3, 4]: coexisting with a psychotic disorder; as a side effect of central stimulants used in the treatment for narcolepsy [15]; or in relation to sensory misperceptions and other narcolepsy symptoms that may form delusions and simulate a psychotic disorder. Several case reports and clinical studies report that hypnagogic and hypnopompic hallucinations can be misinterpreted as the active psychotic state of schizophrenia and lead to misdiagnoses and inappropriate treatment [2–17]. Szücs et al. [6] postulate that there is a subgroup of narcolepsy characterized by frequent hypnagogic/hypnopompic hallucinations and sleepiness in which daytime hallucinations are of convincing nature for the patient, who may be disturbed in his/her reality testing. It is therefore understandable how the differentiation between psychosis and narcolepsy may become problematic. Some good points to help clinicians in the differential diagnosis are personality deterioration and thinking disturbance, which seemed a relevant feature in the case we are discussing. Our patient had clear thought-processing difficulties, with frank formal thought disorder at times, loss of social functioning (i.e., deterioration of academic performance and engagement with peers, with no concerns on these areas prior to his first admission), inappropriate affect and lack of insight into his illness, which all seem to support a diagnosis of schizophrenia with comorbid narcolepsy. However, the fact that some of his symptoms were clearly related to his sleep pattern appeared to indicate that narcolepsy was playing a more causal role in those symptoms. The response to the trial with zopiclone and modafinil proved that approach helpful, as the nonsleep related psychotic symptoms became more evident, making clear that a psychopharmacological approach with antipsychotic medication was appropriate to address them. 

The fact that the diagnosis of narcolepsy that was based on clinical grounds and further testing (i.e., PSG, MSLT) had to be put on hold resulted in some difficulties teasing out which symptoms were due to which conditions. It is not possible to state whether having additional data would have changed our management plan.

In their practice, clinicians can encounter challenging cases with unusual presentations where there may be limited evidence-based knowledge with which to make treatment decisions. In these situations, as our case illustrates, careful consideration and assessment of the clinical picture, history of the symptoms, discussion and consultation with relevant professionals, and subsequent supervised trials of treatment proved a helpful pragmatic approach in making decisions on how to manage a complex presentation.
